# Improvement of Bone Healing by Combined Treatment with Selective Androgen and Estrogen Receptor Modulators in Female and Male Rats

**DOI:** 10.1007/s00223-026-01585-9

**Published:** 2026-08-25

**Authors:** Marina Komrakova, Fabian Nicolai Brunnert, Christian Anders, Kai Oliver Böker, Arndt Friedrich Schilling, Paul Jonathan Roch, Daniel Bernd Hoffmann, Wolfgang Lehmann, Stephan Sehmisch

**Affiliations:** 1 Department of Trauma Surgery, Orthopaedics and Plastic Surgery, University Medical Center Goettingen, Robert-Koch St. 40, 37075 Goettingen, Germany; 2 Department of Trauma Surgery, Hannover Medical School, University of Hannover, Carl-Neuberg-Str. 1, 30625 Hannover, Germany

**Keywords:** Ostarine, Raloxifene, SARM, SERM, Bone healing

## Abstract

With age, both men and women experience a decline of sex-hormones and impaired bone repair. This study examined whether bone healing could be improved by combined treatment with the selective androgen and estrogen receptor modulators ostarine (Ost) and raloxifene (Ral), and compared its efficacy with monotherapy in female and male rat models. Three‑month‑old female (Experiment 1, n = 15/group) and 8‑month‑old male (Experiment 2, n = 15/group) Sprague–Dawley rats were either intact (healthy control) or ovariectomized/orchiectomized (Ovx/Orx). Ovx/Orx rats received daily either no treatment, Ost, Ral, or combined Ost + Ral at the respective monotherapy doses. Eight weeks after Ovx or 12 weeks after Orx, rats underwent bilateral metaphyseal osteotomy of the tibia with plate fixation. Healing was assessed five in females or 6 weeks in males later by micro‑CT, biomechanics, and histology. Increased total, callus, and cortical densities as well as bone volume fraction were observed in the Ost + Ral group, accompanied by reduced callus volume and surface area, indicating advanced healing. The effect of Ost + Ral was stronger than that of Ral, whereas Ost alone did not alter bone parameters. Biomechanical properties were improved in the Ral and Ost + Ral groups relative to Ovx/Orx controls. Uterus weight was lowest in Ovx and Ovx + Ral groups and enhanced by Ost and Ost + Ral. Prostate weight was lowest in Orx and Orx + Ral rats and increased by Ost. The combination with Ral reduced the androgenic effect of Ost on prostate. Combined treatment may represent a promising approach for osteoporotic bone repair in both sexes, though potential side effects of Ost require consideration.

**Supplementary Information:**

The online version contains supplementary material available at https://doi.org/10.1007/s00223-026-01585-9.

## Introduction

The levels of sex hormones decrease with aging, leading to the development of osteoporosis and subsequent bone fractures. Fragility fractures resulting from low-energy trauma represent the main clinical consequence of osteoporosis [7]. In animal studies, osteoporotic bone healing has been associated with decreased callus formation and bone growth, bone mineral density and biomechanical strength [17, 20, 38]. The results of clinical studies also indicate a potential adverse effect of osteoporosis on bone healing, although the evidence remains inconclusive [17].

Sex hormones play an important role in bone repair in both men and women, and there are sex differences in bone healing [15, 40]. Men tend to have faster healing than women but exhibit higher postoperative complications and mortality after fracture, whereas women have a higher risk of non-union and delayed healing [15, 40]. Estrogen acts through two receptors, estrogen receptor-alpha (ER-α) and estrogen receptor-beta (ER-β), which are both found in bone [18]. It induces the release of osteoprotegerin (OPG), which binds to Receptor Activator of NF-κB Ligand (RANKL) and inhibits osteoclast maturation and bone resorption [9]. The skeletal effects of androgens are mediated through androgen receptor (AR) activation by testosterone and its metabolite dihydrotestosterone (DHT), and partially through ERs following aromatization of testosterone to estrogens [32, 52]. Testosterone can also increase the expression of OPG, thereby inhibiting osteoclast maturation [12], whereas DHT reduces OPG in a dose-dependent manner [21, 40].

Commonly prescribed osteoporosis medications are recommended for bone healing, but are not routinely used due to insufficient data on their benefits for fracture treatment [17, 39]. The primary concern associated with hormone therapy is potential side effects [3, 47]. The use of non-steroidal selective androgen or estrogen receptor modulators (SARMs or SERMs) is emerging as a promising approach with fewer side effects for the treatment of osteoporosis and frailty. Ostarine (enobosarm, MK-2866, GTx-024, or S-22) is the most extensively studied SARM with notable therapeutic potential [51]. Raloxifene (Ral), a SERM approved to treat postmenopausal osteoporosis, has been reported to prevent bone loss in men with prostate cancer without feminizing effects [1, 43]. In our recent studies, we have shown a beneficial effect of ostarine (Ost) on bone healing in both female and male rats [27, 29]. Bone healing was improved when Ost was used as osteoporosis prophylaxis, i.e., prior to osteotomy, while administration immediately after osteotomy was associated with negative effects on bone healing in male rats [29]. The influence of Ral on bone healing has been reported with both favorable and unfavorable results [41, 48, 49]. The combination of SARMs and SERMs has been the subject of only a few studies so far, and has shown beneficial effects on osteoporotic bone tissue [16, 23, 25], whereas its effects on bone healing remain unexplored.

In the present study, we hypothesized that combined treatment with Ost and Ral would be favorable for bone healing and superior to single treatments. The primary outcome was bone healing, assessed by biomechanical testing, while secondary outcomes included micro-computed tomography (micro-CT) parameters, gene expression, and histological analyses. The study was conducted in two established rat models representing postmenopausal osteoporosis in females and age-related osteoporosis in males, and also addressed potential sex-specific differences in treatment response. Some data on muscle tissue, intact bone and general parameters of the rat models have been reported previously [23, 25, 44, 45].

## Material and Methods

### General Procedures

**Fig. 1:**
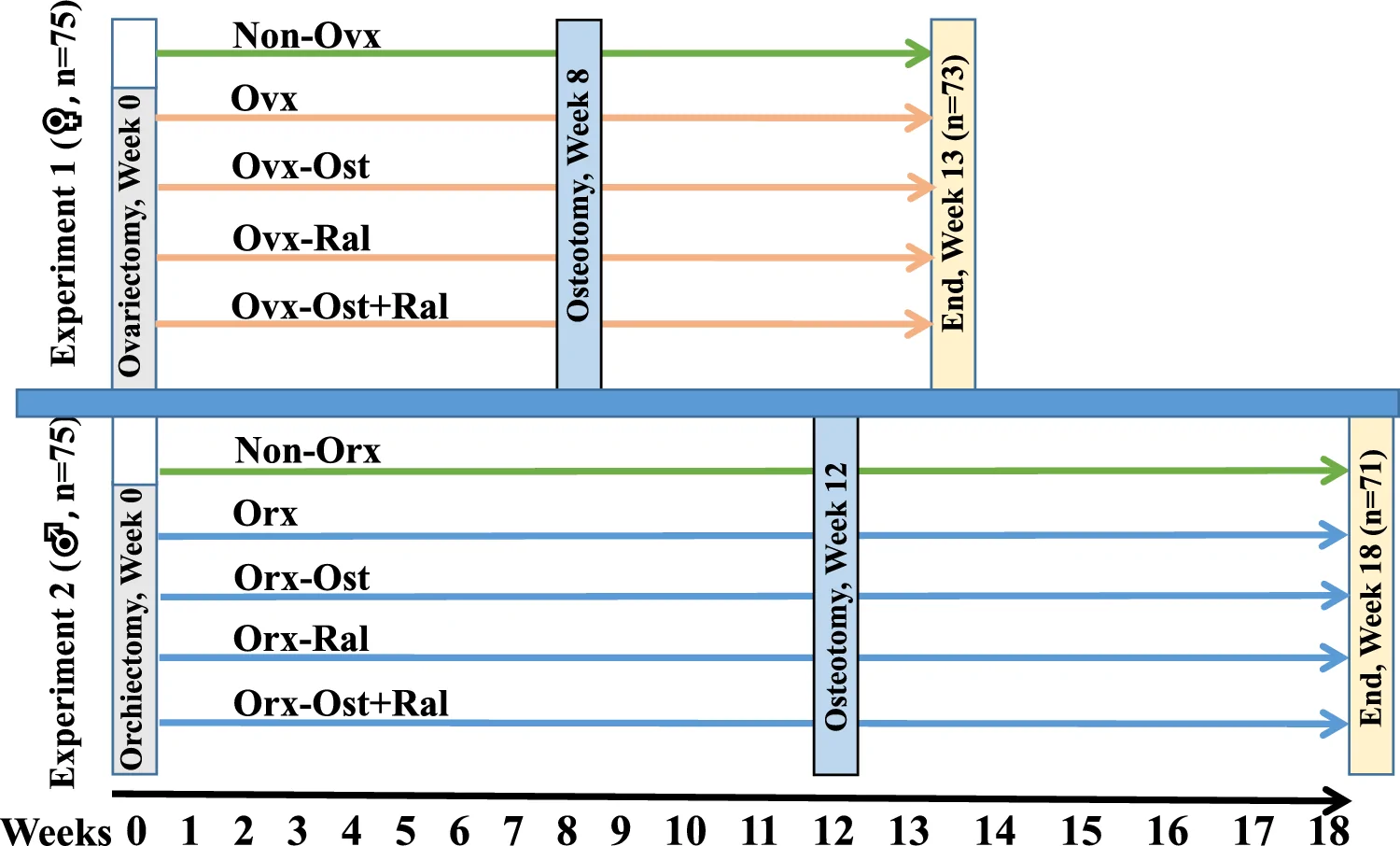
Schematic flowchart of the two experiments. Experiment 1: Non-ovariectomized (Non-Ovx) and ovariectomized (Ovx) rats treated after Ovx with either ostarine (Ost), raloxifene (Ral), or both substances for up to 13 weeks. Experiment 2: Non-orchiectomized (Non-Orx) and orchiectomized (Orx) rats treated after Orx with either Ost, Ral, or the combination Ost + Ral for up to 18 weeks. Bilateral osteotomy was performed at week 8 in Exp. 1 and at week 12 in Exp. 2. Each group initially consisted of 15 rats in both experiments (n = 75/experiment). The final sample sizes (n = 73 in Exp. 1 and n = 71 in Exp. 2) reflect animal losses

Sprague–Dawley rats were obtained from Janvier (Le Genest-Saint-Isle, France). Rats were housed in groups of three or four in standard cages under a 12-h light:12-h dark regime at 22 ± 2 °C. Cage enrichment included cardboard tunnels, wood splinters, straw, and wooden blocks. All rats received a soy-free diet (Ssniff special diet GmbH) and demineralized water throughout the experiment. The animal study protocol was approved by the local regional government (14/1396, Oldenburg, Germany) prior to the study. In total, 150 rats were taken for two experiments (Fig. 1). In both experiments, rats were either gonadectomized or left intact at the beginning of the study and immediately after that they were treated with substances according to the experimental protocol (Fig. 1). Ost (MK-2866, Shanghai Biochempartner Co., Ltd., Shanghai, China) and Ral (Evista, Eli Lilly and Company, Indianapolis, USA) were administered to the rats with the soy-free pelleted diet (Ssniff special diet GmbH, Soest, Germany). The daily doses were 0.4 mg/kg body weight (BW) for Ost and 7 mg/kg BW for Ral [27, 29, 49]. Food intake and BW were recorded weekly.

After 8 (Experiment 1) or 12 (Experiment 2) weeks, bilateral osteotomy of the tibia was performed in all rats. After osteotomy, rats received subcutaneous (s.c.) injections of four fluorescent dyes to label newly formed callus: xylenol orange (XO, 90 mg/kg BW, Meck KGaA, Darmstadt, Germany), calcein green (CG, 10 mg/kg BW, Waldeck GmbH, Münster, Germany), alizarin complexone (AC, 30 mg/kg BW, Merck KGaA, Darmstadt, Germany), and tetracycline hydrochloride (TC, 25 mg/kg BW, Carl Roth GmbH + Co. KG, Karlsruhe, Germany).

At the end of the experiments, blood was collected by cardiac puncture under deep isoflurane anesthesia, and serum was stored at − 20 °C until analysis. The tibiae were dissected free of soft tissue and the osteosynthesis material was removed. One randomly selected tibia was stored at − 20 °C and subjected to the sequential micro-CT, biomechanical, and histological analyses. Metaphyseal bone clips from the contralateral tibiae were immediately stored at − 80 °C for gene expression analysis and could not be used for other analyses.

### ***Experiment 1 (***♀)***. Ost and Ral Treatments in Estrogen-Deficient Female Rats***

Three-month-old female rats were used as a model of postmenopausal osteoporosis. Rats were left intact to serve as a healthy control (Non-Ovx, n = 15) or bilaterally ovariectomized (Ovx, n = 60) (Fig. 1). For Ovx, after placing the rat in the lateral position, the skin was sharply dissected with scissors approximately 1 cm ventral to the spine and 1 cm caudal to the costal arch. Subcutaneous tissue and muscle were bluntly dissected and the ovary was exposed. Below the ovary, the uterine horn was clamped with a ligature clamp, followed by the ligation with Vicryl® 4.0 sutures (Ethicon; Johnson & Johnson Medical GmbH, Norderstedt, Germany) and sharp separation of the ovary just above the ligature. Finally, the peritoneum and muscle layer were closed with Vicryl® 4.0 sutures and the skin was stapled with Michel brackets (7.5 × 1.75 mm; Gebrüder Martin GmbH & Co. KG, Tuttlingen, Deutschland). The wound was treated with Braunol® solution (B. Braun Melsungen AG, Melsungen, Germany). On the contralateral site the same procedure was repeated [54]. After surgery, Ovx rats were divided into four groups (15 rats each): 1) untreated osteoporotic control (Ovx), 2) rats given Ost (Ovx‑Ost), 3) rats receiving Ral (Ovx‑Ral), and 4) rats treated with Ost and Ral (Ovx‑Ost + Ral) (Fig. 1).

The average daily doses, calculated based on the average daily food intake of the rats, were for Ost 0.37 ± 0.05 and 0.40 ± 0.08 mg/kg BW in the Ovx-Ost and Ovx-Ost + Ral groups and for Ral 6.65 ± 1.15 and 7.9 ± 4.58 mg/kg BW in the Ovx-Ral and Ovx-Ost + Ral groups, respectively [23, 44]. After 8 weeks of treatments, bilateral osteotomy of the tibia was performed in all rats and the treatments were continued for up to 5 weeks after osteotomy (Fig. 1). Fluorescent dyes were applied as follows: XO on day 12, CG on day 19, AC on day 27, and TC on day 35 after osteotomy, respectively [27].

A total of two animals were lost during the experimental period due to anesthetic or surgical complications (Non-Orx: n = 1; Ral: n = 1).

### ***Experiment 2 (***♂)***. Ost and Ral Treatments in Androgen-Deficient Male Rats***

Eight-month-old male rats were used as a model of age-related osteoporosis in males. Rats were non-orchiectomized (Non-Orx, n = 15) or bilaterally orchiectomized (Orx, n = 60). For orchiectomy, rats were placed supine and the scrotal skin and scrotum with tunica were opened. The testicle, surrounding blood vessels, fat and connective tissue were exposed and clamped with a ligature, followed by Vicryl® 4.0 suture ligation and sharp separation of the testicle. To avoid bleeding, small surrounding vessels were cauterized (C300 Cautery Handle, Merlin Medical, Lemgo, Germany). The incision was closed with Vicryl® 4.0 suture for the scrotum and 4.0 Prolene suture (Ethicon, Johnson & Johnson, Norderstedt, Germany) for the skin. The surgical area was then disinfected with Braunol® solution. The same procedure was performed on the contralateral side [37]. After surgery, Orx rats were divided into 4 groups (15 rats each) and treated according to the experimental design (Fig. 1) as follows: 1) untreated osteoporotic control (Orx), 2) rats treated with Ost (Orx-Ost), 3) rats given Ral (Orx-Ral), 4) rats administered both Ost and Ral (Orx–Ost + Ral).

The daily doses for Ost were 0.52 ± 0.08 and 0.58 ± 0.11 mg/kg BW in Orx-Ost and Orx–Ost + Ral groups, and for Ral 10.7 ± 2.1 and 11.6 ± 2.2 mg/kg BW in Orx-Ral and Orx–Ost + Ral groups, respectively [25, 45]. After 12 weeks of treatments, a bilateral tibial osteotomy was conducted in all rats and treatments continued for up to 6 weeks after osteotomy (Fig. 1). Fluorescent dyes were applied as follows: XO on day 12, CG on day 22, AC on day 32, and TC on day 42 after osteotomy, respectively [29].

A total of four animals were lost during the experimental period due to anesthetic or surgical complications (Non-Orx: n = 1; Orx: n = 1; Orx–Ost + Ral: n = 2).

### Osteotomy Surgery

**Fig. 2:**
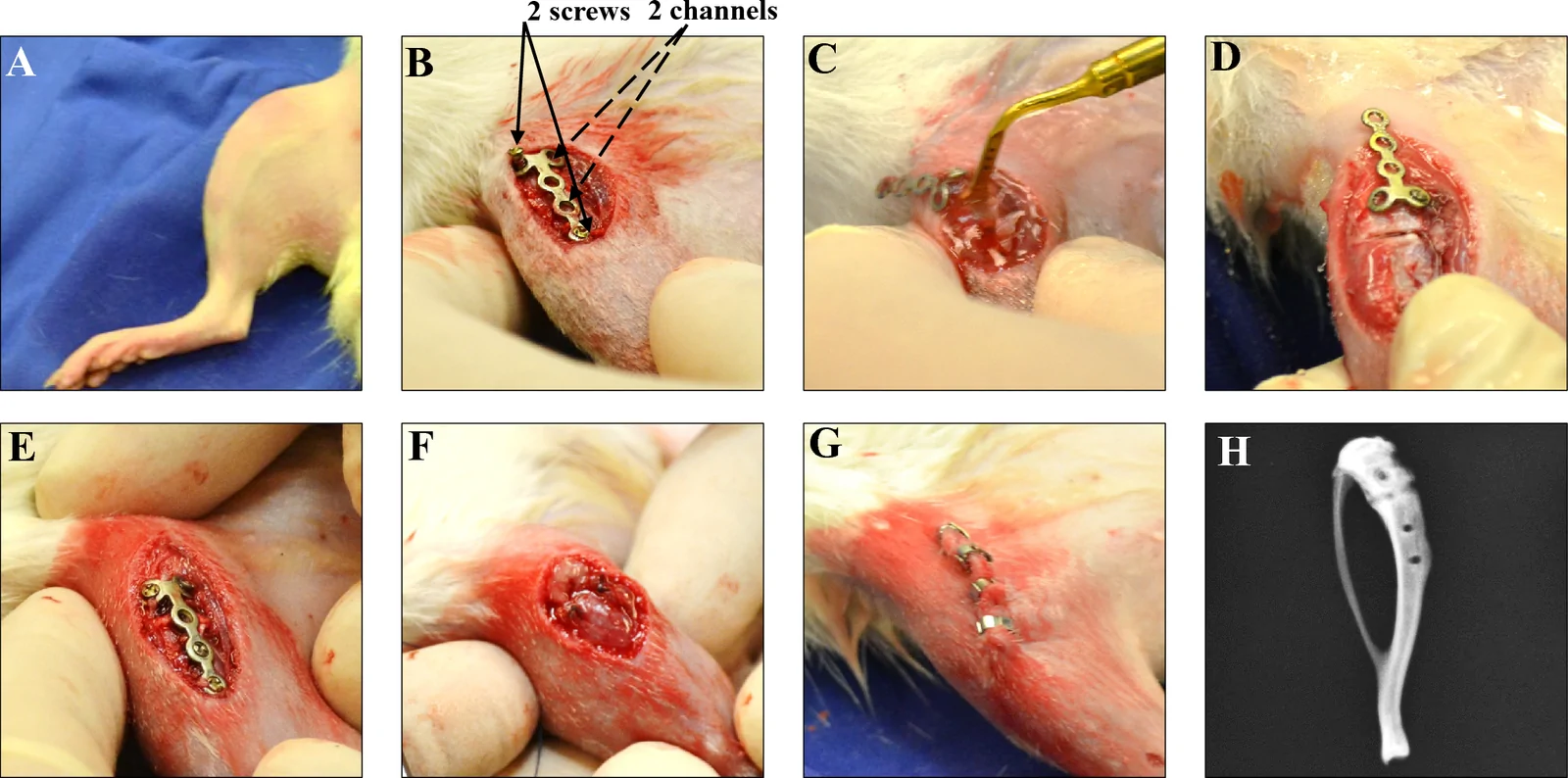
Osteotomy of the tibia metaphysis with osteosynthesis. **a** Shaved and disinfected surgical site, **b** drilling of proximal and distal holes, fixation of T-plate with two screws (continuous arrows) and drilling of other proximal and distal holes (dashed line arrows), **c** removing the distal screw, moving the plate to the side and sawing the tibia metaphysis, **d** 0.5 mm gap, **e** fixation of osteotomized bone ends with plate and four screws; **f** stitching the muscle tissue, **g** stapling the skin with wound brackets, **h** radiography of tibia after removal of plate and screws at the end of experiment (bone sample from Exp. 1)

All rats underwent bilateral tibial osteotomies [26, 49] at week 8 post-ovariectomy in female rats and at week 12 post-orchiectomy in male rats. The skin of the proximal tibia was opened and the muscle was pushed aside (Fig. 2A–H). A five-hole T-shaped titanium plate (XS, 57-05140, Stryker Trauma, Selzach, Switzerland) was fixed to the anteromedial surface of the tibia with one proximal (7 mm length) and one distal (4 mm length) screws (Stryker Trauma). The other two channels were drilled and initially left without screws (Fig. 2B). To perform the osteotomy, the distal screw was removed and the plate was moved laterally (Fig. 2C, D). The osteotomy was performed 7 mm distal to the knee surface using a pulsed ultrasound saw (Piezosurgery, Mectron Medical Technology, Carasco, Italy). Finally, the plate was repositioned and fixed with 4 screws (Fig. 2E). The length of the additional screws was 6 mm for proximal and 5 mm for distal. The incision was closed with Vicryl 4.0 suture (Johnson & Johnson (Ethicon), Norderstedt, Germany) for muscle and with Michel wound brackets for skin (7.5 × 1.75 mm, Gebrüder Martin GmbH & Co. KG, Tuttlingen, Germany) (Fig. 2F, G).

### General Pre- and Post-operative Treatments

The rats received s.c. 5 mg/kg BW carprofen (Rimadyl® 50 mg/ml, Zoetis, Berlin, Germany) 30 min before surgeries. Thereafter, the rats were anesthetized with 3% isoflurane (Forene® 100%, B506, 250 ml, AbbVie GmbH, Wiesbaden, Germany) and an Iso-transponder (UNO, 6900 AA Zevenaar, Netherlands) was implanted s.c. in the neck region during ovariectomy or orchiectomy surgery. To prevent corneal desiccation, the Corneregel® eye gel (Bausch & Lomb, Berlin, Germany) was applied during surgery. The surgical site was shaved using an electric clipper (Aesculap Suhl GmbH, Suhl, Germany) and disinfected with Kodan® (Schülke & Mayr GmbH, Norderstedt, Germany) (Fig. 2a). Postoperatively, each animal received 3 ml of warm isotonic saline s.c. to prevent dehydration. Animals were then placed in a cage with heating pads and monitored continuously until full recovery [54].

For the osteotomy surgery, rats were additionally anesthetized with an intraperitoneal (i.p.) injection of 38 mg ketamine (Ketamin Inresa, Inresa Arzneimittel GmbH, Freiburg, Germany) and 2.5 mg midazolam (Rotexmedica GmbH, Trittau, Germany) per kg BW. These osteotomized animals received a single s.c. dose of enrofloxacin (Bayer, Leverkusen, Germany) prior to surgery as antimicrobial prophylaxis, followed by daily s.c. injections of carprofen (5 mg/kg/day) for the first three postoperative days.

### Serum Analysis

Alkaline phosphatase (AP) was measured in serum (n = 8 per group) at the Department of Clinical Chemistry, University Medical Center, Goettingen, Germany, using an automated chemistry analyzer (Architect c16000 analyzer, Abbott, Wiesbaden, Germany) and the corresponding alkaline phosphatase reagent kit (7D55, Abbott). From the same samples (n = 8 per group), osteocalcin (OC) and Cross Linked C-telopeptide of Type I Collagen (CTX-I) were analyzed in our laboratory using commercial enzyme immunoassay kits (rat-MID™ Osteocalcin EIA and RatLaps (CTX-I) EIA; Immunodiagnostic Systems GmbH, Frankfurt am Main, Germany) according to the manufacturer’s instructions [25, 29].

### pQCT Analysis of Abdominal CSA and Spine

Prior to osteotomy surgery and at the end of experiment, rats (n = 5 per group) were isoflurane-anesthetized and scanned using an in-vivo peripheral quantitative computed tomography device (pQCT, XCT Research SA, Stratec Medizintechnik GmbH, Pforzheim, Germany). The fourth lumbar vertebral body (L4) with abdomen was scanned using the following scan protocol: 90 mm measurement diameter, 0.2 mm voxel size, 90 s scan time, 0.3 mA anode current, 50 kV high voltage, 180 projections, and 1° angle between detectors. The bone mineral density (BMD, mg/cm^3^), stress–strain-index (SSI), and abdominal cross sectional area (CSA, mm^2^) were assessed with the aid of the XCT-6.20C software (Stratec Medizintechnik GmbH) [30].

### Micro-CT Analysis

To evaluate structural parameters, one randomly selected tibia per animal was scanned with a Quantum FX micro-CT (Caliper Sciences, Hopkinton, MA, USA). The number of analyzed animals ranged from 14 to 15 per group in Experiment 1, and from 13 to 15 per group in Experiment 2. The scans were performed using the following protocol: 70 kVp, 200 μA, 2-min exposure time, 360° rotation, 3600 projections, 20 × 20 mm^2^ field of view, 512 pixel matrix, and 40 × 40 × 40 μm^3^ effective voxel size [29]. A phantom block of five hydroxyapatite elements of different mineral densities was scanned with each tibia to convert the data to bone mineral density (g/cm^3^). The scans were analyzed using a program developed in our laboratory. A later version of this program is Scry v6.0 software (Kuchel and Sautter UG, Bad Teinach-Zavelstein, Germany) [2]. The measurement area extended 2.25 mm proximally and distally from the osteotomy line. In each experiment, mean thresholds for total bone, cortical bone, calcified callus, and soft callus were visually determined using three osteoporotic control rats and three healthy control rats and applied to all bones examined within the respective experiment (Supp. Figure 1A–H). The following parameters were evaluated: total bone density and volume (Tt. BMD and Tt. V), calcified callus density and volume (Calcif. callus BMD and Calcif. callus V), cortical density and volume (Ct. BMD and Ct. V), soft callus density and volume (St. BMD and St. V), and bone volume fraction (BV/TV) [8, 26, 29].

### Biomechanical Analysis

After micro-CT analysis, a three-point bending test was conducted in the same tibiae (Experiment 1: n = 14–15 per group; Experiment 2: n = 14–15 per group) using a Zwick/Roell testing device (type 145660 Z020/TND, Ulm, Germany), as previously described in detail [31]. To allow for the subsequent usage of the tibia in histomorphometrical analyses, a non-destructive mechanical testing protocol was applied. The tibia was loaded at the osteotomy line at the tibial tuberosity at a feed motion rate of 5 mm/min. The test was automatically terminated by the software (TestXpert, Zwick/Roell) as soon as a force drop of more than 2 N was detected, thereby preventing the onset of plastic deformation and subsequent fracture of the bone. The stiffness (N/mm), defined as the slope of the linear rise of the curve during elastic deformation, and the yield load (N), defined as the end point of elastic deformation, were calculated using MS Excel (MS Office 2016) [31].

### Histomorphometrical Analyses

Following biomechanical analysis, the tibiae (Experiment 1: n = 14–15 per group; Experiment 2: n = 13–15 per group) were embedded in methyl methacrylate (Merck, Darmstadt, Germany). Thereafter, 150-µm-thick longitudinal sections of the tibiae were cut using a diamond saw microtome (Leica SP1600, Leica Instruments GmbH, Nussloch, Germany) [29]. The time of the first osseous bridging of the osteotomized bone was determined visually using a microscope (Leica, Leitz DM RXE, Bensheim, Germany) by analyzing all bone sections (at least ten) [28]. Three central histological sections were chosen to measure the total area (Tt.Ar. and labeling-specific area (CG, AC, and TC of the callus using a digital camera (Leica DC300F and a zoom stereo microscope (Leica MZ75. The measurement area of the tibia was 2.25 mm proximally and distally from the osteotomy line. Three regions were identified: ventral, plate side (v,dorsal, opposite site (d; and (e endosteal part [27, 29]. The sections (n = 3) of the tibia were microradiographed using a Faxitron Cabinet X-ray system (Hewlett-Packard, Buffalo Grove, IL, USA) and Kodak Industrex film (SR45, 100 NIF, Kodak, Paris, France). Microradiographs were analyzed similar to the histological sections. Within the measurement area, periosteal callus width (Cl.Wi.v, Cl.Wi.d) and density (Cl.Dn.v, Cl.Dn.d), endosteal callus density (Cl.Dn.e) were measured in microradiographs according to the nomenclature described in Dempster et al. [13].

### Gene Expression Analysis

Tibia samples (n = 5 per group) were homogenized using a Micro-Dismembrator S (Sartorius, Goettingen, Germany), and total RNA was extracted using the TRIzol reagent according to the manufacturer`s protocol (Thermo Fischer Scientific, WA, USA). RNA concentration was measured with the aid of a photometer (DeNovix DS-11 FX + System, DeNovix, NC, USA) and 1000 ng RNA were reverse transcribed using iScript cDNA Synthesis Kit (Bio-Rad, Hercules, CA, USA) [23]. Expression of rat genes was determined by quantitative real-time polymerase chain reaction (qPCR) based on SYBR Green detection using an iCycler (CFX96, Bio-Rad Laboratories). Ready-to-use primer pairs were obtained from Qiagen (QuantiTect Primer Assays, Qiagen, Hilden, Germany). The expression of the following genes was measured: alkaline phosphatase (Ap), osteocalcin (Oc), receptor activator of nuclear factor kB ligand (Rankl), osteoprotegerin (Opg), androgen receptor (Ar), estrogen receptor α and β (Er-α and Er-β). Expression of Er-β was below detectable levels and is therefore not shown in the results. Relative gene expression was calculated using 2^−ΔΔCT^ method [35] for each gene of interest, where ΔΔCT was calculated as follows: (CT experimental group gene—CT reference gene β-2 microglobulin (MG))—(CT Non-Ovx/Non-Orx group—reference gene β-2 MG).

### Statistical Analyses

Statistical analyses were performed with SAS (version 9.1; SAS Institute, Cary, NC, USA) and GraphPad Prism (version 5.04; GraphPad Software, San Diego, CA, USA). Normality was evaluated using the Kolmogorov–Smirnov, D’Agostino–Pearson, and Shapiro–Wilk tests. For normally distributed data, group differences were analyzed using one-way ANOVA followed by Tukey’s post hoc test. For non-normally distributed data, the Kruskal–Wallis test followed by Dunn’s multiple-comparison test was applied. A p-value less than 0.05 was considered statistically significant.

## Results

### *Experiment 1(*♀)*. Ost and Ral Treatments in Estrogen-Deficient Female Rats*

#### Animal Model

BW of rats increased after Ovx from week 1 onward. Ost treatment caused further BW increase, whereas Ral and Ral + Ost treatments maintained it at the level of Non-Ovx rats (Table 1, Suppl. Figure 2A). In general, rat in the Ovx and Ovx-Ost groups consumed more food than the rats in the other groups (Suppl. Figure 2B). Uterus weight was significantly lower in the Ovx and Ovx-Ral groups than in the other 3 groups (Table 1).

**Table 1:** Experiment 1 (♀). Final body weight, serum parameters, biomechanical test and gene expression in tibia callus of Non-Ovx and Ovx rats either treated with ostarine (Ost), raloxifene (Ral) or combined treatment (Ost + Ral)

Parameters	Non-Ovx		Ovx		Ovx-Ost		Ovx-Ral		Ovx-Ost + Ral	
	Mean	SD	Mean	SD	Mean	SD	Mean	SD	Mean	SD
*Weights (n* = *14–15/group)*										
Final body weight (g) #Δ	342^**bc**^	17	452	31	495^**b**^	41	330^**bc**^	14	356^**bc**^	17
Uterus weight (g) #Δ	0.75^**bd**^	0.19	0.22	0.07	0.62^**bd**^	0.09	0.25	0.05	0.66^**bd**^	0.16
*Serum (n* = *8/group)*										
AP (U/l)Δ	135^**c**^	38	158	47	220^**b**^	73	180	36	190^**a**^	49
OC (ng/ml) Δ	334	112	385	62	357	103	310	124	205^**abc**^	41
CTX-I (ng/ml)Δ	18.5^**bc**^	3.6	27.4	3.6	27.1	8.1	21.0	5.5	21.7	6.3
*Biomechanical test (n* = *14–15/group)*										
Stiffness (N/mm)	114.9	58.6	62.1	21.6	96.8	46.2	143.8^**b**^	54.3	136.3^**b**^	50.0
Yield load (N)	110.6^**b**^	45.4	49.7	25.2	72.2	40.4	73.3	35.3	57.2	17.0
*Micro-CT (n* = *14–15/group)*										
Tt. BMD (mg/cm^3^)	658^**bc**^	67	559	45	534	44	642^**bc**^	40	735*	53
Tt. V (mm^3^)	93^**bc**^	10	119	28	126	20	94^**c**^	18	81^**bc**^	12
Calcif. callus BMD (mg/cm^3^)	783^**bcd**^	38	745	17	746	22	747	30	780^**bcd**^	16
Caclif. callus V (mm^3^)	41	8	46	13	49	10	39	10	31^**bc**^	6
Ct. BMD (mg/cm^3^)	1296^**c**^	9	1280	17	1267	16	1308^**bc**^	9	1312^**bc**^	14
Ct. V (mm^3^)	20	3	19	3	17	3	19	2	23^**bcd**^	3
St. V (mm^3^)	33^**bc**^	7	54	15	60	14	36^**bc**^	8	26^**bc**^	5
BV/TV (%)	64^**bc**^	7	55	4	53	5	63^**bc**^	2	68^**bc**^	5
*Gene expression (n* = *5/group)*										
Opg	1.1^**b**^	0.5	2.2	0.9	1.7	0.6	2.6^**ac**^	0.4	3.2^**abc**^	1.1
Rankl	1.2*	0.6	7.6	4.5	6.0	1.2	5.2	0.8	7.0	2.4
Opg/Rankl	1.1^**bc**^	0.5	0.5	0.4	0.3	0.2	0.5	0.1	0.5^**a**^	0.1
Oc	1.1^**bc**^	0.4	2.2	0.9	1.8	0.7	1.1^**b**^	0.3	0.9^**bc**^	0.3
Alp	1.1*	0.7	3.5	2.0	3.2	0.4	3.7	1.0	5.0^**c**^	1.3
Er-α	1.2^**c**^	0.7	1.9	0.9	2.6	1.7	2.1	0.7	1.8	0.6
Ar	1.1^**b**^	0.5	2.8	1.5	2.4	1.4	3.3^**a**^	1.1	3.7^**ab**^	1.2
*Histology (n* = *14–15/group)*										
Osteotomy bridging (day) 21		6	20	5	25	7	20	2	17^**c**^	4

^a^differs from Non-Ovx, ^b^differs from Ovx, ^c^differs from Ovx-Ost, ^d^differs from Ovx-Ral

*differs from all other groups (P < 0.05, Dunn’s test: serum OC, Yield load, St.V, Opg/Rankl, Oc, Alp, ER-α, AR, Tukey-test: all other data). # data were published in [44], Δ data were published in[23]

Serum activity of AP was higher in the Ovx-Ost group than in the Non-Ovx and Ovx groups. Combined Ost + Ral treatment increased AP level compared to the Non-Ovx group (Table 1). Serum OC was lower in the Ovx-Ost + Ral group than in the Non-Ovx, Ovx and Ovx-Ost groups (Table 1). Serum CTX-I was higher in the Ovx and Ovx-Ost groups than that in the Non-Ovx group (Table 1).

**Fig. 3:**
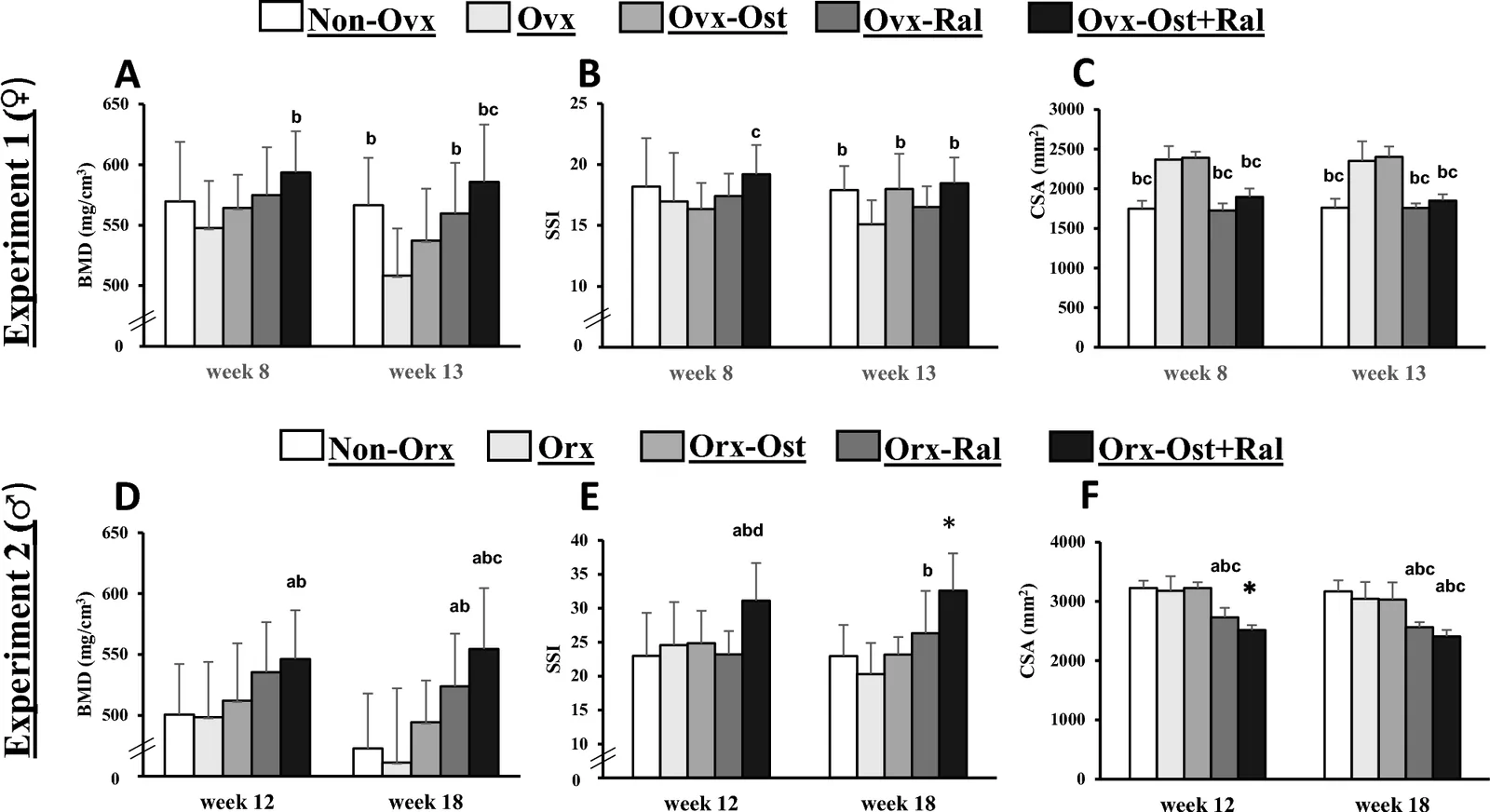
pQCT analysis of spine and abdominal cross sectional area (CSA) (n = 5/group). The analysis was conducted prior to osteotomy surgery (week 8 or 12) and at the end of experiment (week 13 or 18) (n = 5 per group). (*) differs from all other groups, **a** differs from Non-Ovx/Non-Orx, **b** differs from Ovx/Orx, **c** differs from Ost, **d** differs from Ral, **e** differs from Ost + Ral (*p* < 0.05, Dunn´s test: C, E,F; Tukey test: A,B,D). BMD: bone mineral density, SSI: stress–strain-index. BMD, SSI and CSA at week 18 (D-F) have been published in [45]

pQCT analysis of L4 revealed higher BMD in the Ovx-Ost + Ral group than in the Ovx group, and higher SSI than in the Ost group at week 8 (Fig. 3A,B). At week 13, BMD and SSI were higher in Non-Ovx and Ovx-Ost + Ral groups than in the Ovx group. BMD was additionally higher in the Ovx-Ost + Ral group than in the Ost group, while BMD in the Ral group and SSI in the Ost group were higher compared with the Ovx group (Fig. 3A, B). CSA was larger in the Ovx and Ovx-Ost groups than in the other 3 groups at weeks 8 and 13 (Fig. 3C).

#### Bone Healing Analyses

Biomechanical testing revealed a diminished yield load in Ovx rats, whereas stiffness was increased after Ral and Ost + Ral treatments (Table 1).

Micro-CT analysis showed that Ovx reduced Tt. BMD, Calcif. callus BMD and BV/TV at the osteotomy site and enhanced Tt. V and St. V compared to the Non-Ovx rats (Table 1). Ost treatment did not change the effect of Ovx on these parameters (Table 1). Ral increased Tt. BMD, Ct. BMD and BV/TV and decreased St.V in the Ovx rats. Under combined treatment of Ost + Ral, all density parameters (Tt.BMD, Calcif. callus BMD, Ct. BMD) and Ct.V and BV/TV were increased, whereas other volumetric parameters (Tt.V, Calcif. callus V, and St.V) were decreased (Table 1). St. BMD was not significantly different between the treatment groups (data not shown).

**Fig. 4:**
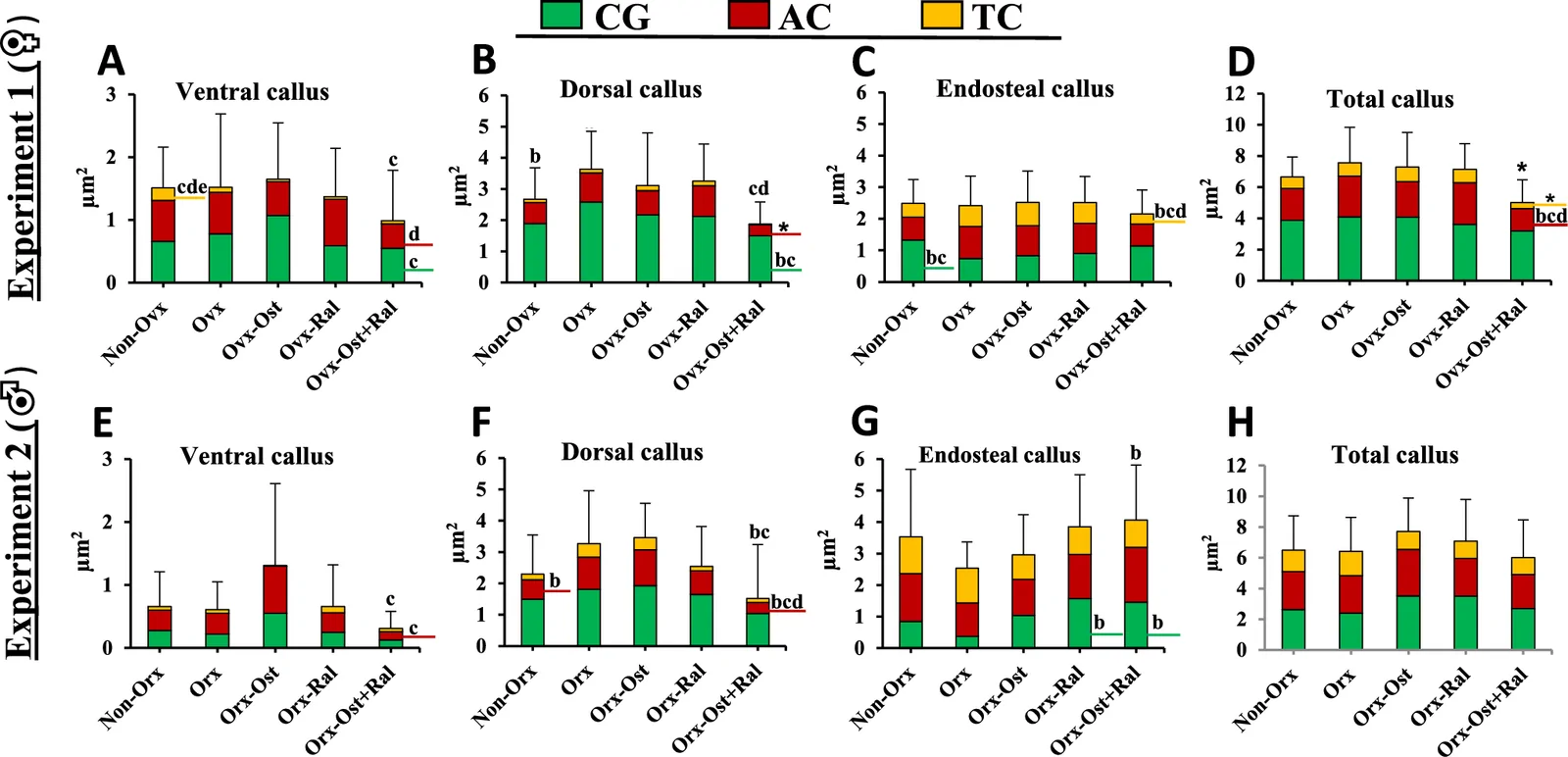
Callus area (µm^2^) divided into ventral **A**, **E**, dorsal **B**, **F**, and endosteal **C**, **G** regions and total callus area **D**, **H** measured at the sections of tibia according to the fluorescence-labeled areas [xylenol orange and calcein green (CG), alizarin complexone (AC), and tetracycline (TC)] (Experiment 1: n = 14–15/group; Experiment 2: n = 13–15/group). (*) differs from all other groups, (a) differs from Non-Ovx/Non-Orx, (b) differs from Ovx/Orx, (c) differs from Ost, (d) differs from Ral, (e) differs from Ost + Ral (p < 0.05, Dunn´s test: A,C,E,G; Tukey test: all other data)

Fluorescence-labeled area (Fig. 4A–D) of total dorsal callus was larger, whereas CG-labeled endosteal callus area was smaller in the Ovx rats compared to the Non-Ovx group (Fig. 4B, C). Under Ost treatment, CG-labeled endosteal callus and TC-labeled ventral callus were smaller than in the Non-Ovx group (Fig. 4A,C). In the Ral group, TC-labeled ventral callus was also smaller compared to the Non-Ovx group (Fig. 4A). The combined Ost and Ral treatment reduced the dorsal areas of CG and AK callus, the endosteal area of TC callus, the total areas of AC and TC callus, and the total area of callus (Fig. 4B,D). Osteotomy bridging was observed earlier in the Ovx-Ost + Ral group than in the Ovx-Ost group (Table 1).

**Fig. 5:**
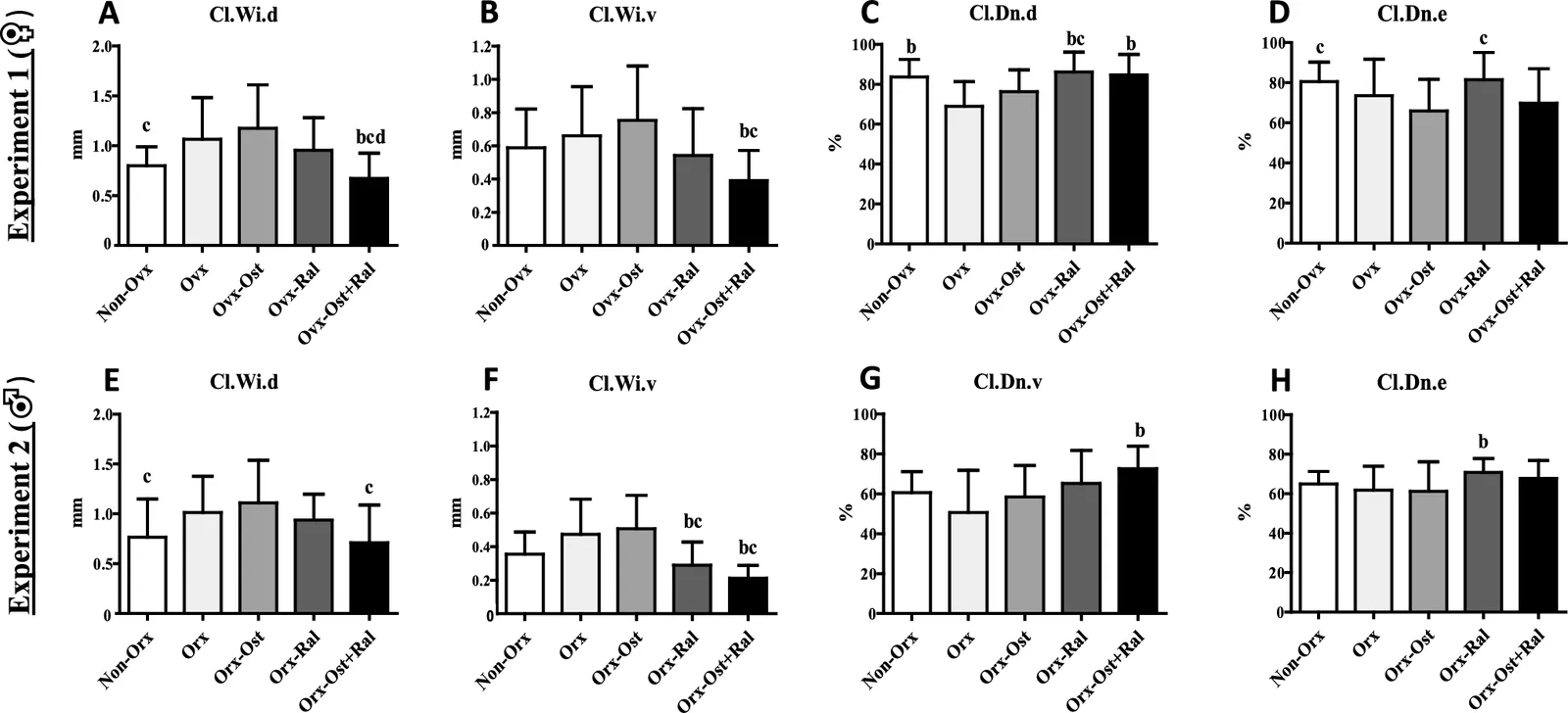
Analysis of microradiographs in Experiment 1 **A**–**D** and Experiment 2 **E**–**H** measured at the osteotomy site at the end of experiments (Experiment 1: n = 14–15/group; Experiment 2: n = 13–15/group). (*) differs from all other groups, (a) differs from Non-Ovx/Non-Orx, (b) differs from Ovx/Orx, (c) differs from Ost, (d) differs from Ral, (e) differs from Ost + Ral (p < 0.05, Dunn´s test: A-C, H; Tukey test: D, E–G). Cl.Wi.d: callus width dorsal, Cl.Wi.v: callus width ventral, Cl.Dn.d: callus density dorsal, Cl.Dn.v: callus density ventral, Cl.Dn.e: callus density endosteal

Analysis of microradiographs (Fig. 5 A-D) revealed diminished Cl.Dn.e after Ovx (Fig. 5C). Ost treatment did not alter callus parameters in Ovx rats and increased Cl.Wi.d and decreased Cl.Dn.e compared to Non-Ovx rats. Ral treatment increased Cl.Dn.d in Ovx rats, whereas Ral + Ost treatment lowered Cl.Wi at the dorsal and ventral aspects and enhanced Cl.Dn.d (Fig. 5A-D).

Gene expression of Opg, Rankl, Oc, Alp and Ar was higher in the Ovx group than in the Non-Ovx group, whereas the Opg/Rankl ratio was lower (Table 1). Gene expression under Ost treatment did not differ from that in Ovx rats (Table 1). Under Ral treatment, Oc gene expression was lower than in the Ovx rats (Table 1). Ost + Ral treatment increased the expression of Opg and Ar genes and decreased Oc expression compared to Ovx rats (Table 1).

### Experiment 2 (♂). Ost and Ral Treatments in Androgen-Deficient Male Rats

#### Animal Model

BW decreased after Orx compared with Non-Orx animals, reaching significant differences from week 14 until the end of the study (Table 2, Suppl. Figure 2C). Ost treatment did not affect BW, whereas under Ral and Ost + Ral treatments it was significantly lower than in other groups after week 2 onward (Table 2, Suppl. Figure 2C). Food intake was lower in Orx group than in the Non-Orx group at the begging and end of the study (Suppl. Figure 2D). Under Ral and Ost + Ral treatments, the rats consumed less food than those in the Orx and Orx–Ost groups during first 6 weeks (Suppl. Figure 2D). Thereafter, food intake in the Orx–Ost + Ral group increased to the level measured in the other groups, whereas it remained at the significant lower level in the Orx + Ral group throughout the study (Suppl. Figure 2A). Orx reduced the prostate weight, while Ost increased it in the Orx-Ost (Table 2). Under Orx–Ost + Ral treatment, prostate weight was higher than in the Ral group (Table 2).

**Table 2:** Experiment 2 (**♂)**. Final body weight, serum parameters, biomechanical test and gene expression in tibia callus of Non-Orx and Orx rats either treated with ostarine (Ost), raloxifene (Ral) or combined treatment (Ost + Ral)

Parameters	Non-Orx		Orx		Orx-Ost		Orx-Ral		Orx–Ost + Ral	
	Mean	SD	Mean	SD	Mean	SD	Mean	SD	Mean	SD
*Weights (n* = *13–15/group)*										
Final body weight (g) + ˄	695^**b**^	34	636	42	669	50	565^**abc**^	47	557^**abc**^	30
Prostate weight (g) + ˄	1.27*	0.38	0.24	0.06	0.63^**bd**^	0.22	0.22	0.10	0.46^**d**^	0.11
*Serum (n* = *8/group)*										
AP (U/l) + ˄	147	59	138	28	165	45	148	39	200^**ab**^	52
OC (ng/ml)˄	121	36	125	46	86	33	90	37	41^**ab**^	16
CTX-I (ng/ml)˄	7.1	1.3	7.7	1.7	6.4	1.7	5.0^b^	0.8	4.5^ab^	1.4
*Biomechanical test (n* = *13–15/group)*										
Stiffness (N/mm)	152.8^**bc**^ 27.7	43.6	22.3	53.9	31.9	120.2^**bc**^	41.9	114.4^**b**^	36.1	
Yield load (N)	62.3^**b**^	17.0	18.9	2.8	38.2	22.5	69.1^**b**^	36.0	54.8	16.0
*Micro-CT (n* = *13–15/group)*										
Tt. BMD (mg/cm^3^)	548	57	511	62	554	88	628^**ab**^	66	646^**abc**^	65
Tt. V (mm3)	134	22	131	30	127	32	122	27	107^**a**^	19
Calcif. callus BMD (mg/cm^3^)	549	18	537	26	550	18	563^**b**^	16	567^**b**^	14
Calcif. callus V (mm^3^)	52	14	50	16	48	14	48	17	39	11
Ct. BMD (mg/cm^3^)	1223	23	1234	25	1235	27	1242	33	1247	20
Ct. V (mm^3^)	33	4	28	4	31	4	36^**bc**^	3	35^**b**^	6
St. V (mm^3^)	49	12	53	15	48	20	38	13	33^**ab**^	9
BV/TV (%)	63	7	60	6	63	8	69^**b**^	6	69^**b**^	7
*Gene expression (n* = *5/group)*										
Opg	1.0	0.2	1.6	0.8	3.8*	2.5	1.4	0.5	1.1	0.3
Rankl	1.0^**b**^	0.3	1.6	0.7	1.1	0.6	1.2	0.4	0.8	0.2
Opg/Rankl	1.0	0.2	1.6	0.8	3.8*	2.5	1.4	0.5	1.1	0.3
Oc	1.1	0.5	1.9	0.6	1.3	0.8	1.5	0.9	1.5	0.5
Alp	1.1	0.5	2.4	1.2	4.5*	2.9	1.3	0.4	1.0^**b**^	0.1
Er-α	1.0	0.2	1.5	0.3	3.6^**ab**^	1.1	4.5^**ab**^	2.0	2.8^**abd**^	0.7
Ar	1.0	0.2	1.2	0.4	3.5^ab^	1.2	4.2^ab^	1.1	2.7^abd^	0.7
*Histology (n* = *13–15/group)*										
Osteotomy bridging (day) 26		5	27	4	26	6	26	5	23	9

^a^differs from Non-Orx, ^b^differs from Orx, ^c^differs from Orx-Ost, ^d^differs from Orx-Ral

*differs from all other groups (P < 0.05, Dunn´s test: Yield Load, Tt.V, Calcif. Callus BMD, St.V, Oc gene expression, Tukey-test: all other data). + Data were published in [45], ˄ data were published in[25]

Serum AP was higher and OC and CTX-I were lower in the Orx–Ost + Ral group than those in the Non-Orx and Orx groups (Table 2). CTX-I in Orx-Ral group was also lower compared to that in the Orx group (Table 2).

pQCT analysis showed that both BMD and SSI of L4 were higher in the Orx–Ost + Ral group than in the Non-Orx and Orx groups at week 12 and compared to Orx-Ost at week 18 (Fig. 3D, E). SSI was the highest under Ost + Ral treatment at week 18 (Fig. 3E). Ral treatment enhanced BMD compared to the Non-Orx and Orx groups and SSI in comparison to the Orx group at week 18 (Fig. 3D, E). CSA was lower in the Orx-Ral and Orx–Ost + Ral groups than in the other 3 groups at both time points studied (Fig. 3F). Data at week 18 have been published previously [45].

#### Bone Healing Analyses

Stiffness and yield load at the osteotomy site were lower in the Orx group compared to the Non-Orx group (Table 2). Ost did not improve these parameters, whereas Ral significantly increased them. Ost + Ral treatment enhanced stiffness of the callus (Table 2).

Micro-CT analyses at the osteotomy site detected no differences in bone parameters in the Orx and Orx-Ost groups compared to the Non-Orx (Table 2). Under Ral treatment, Tt.BMD, Cacif. callus BMD, Ct.V and BV/TV were higher than in the Orx group. In the Orx–Ost + Ral group, Tt.BMD, Calcif. callus BMD, Ct. V and BV/TV were higher, whereas St.V was lower than those in the Orx group (Table 2). St. BMD was not significantly different between the treatment groups (data not shown).

AC-labeled area of dorsal callus was increased in the Orx rats compared to the Non-Orx rats, whereas callus areas in Ost-treated rats did not differ from Non-Orx and Orx groups (Fig. 4E–H). Ral and Ost + Ral treatments resulted in an increase in CG endosteal callus areas (Fig. 4G). AC and total callus areas at the dorsal aspect were smaller and total endosteal callus area was larger in the Orx–Ost + Ral group than in the Orx group. (Fig. 4F, G). Osteotomy bridging was early in Ost + Ral group, though this did not reach statistical significance (Table 2).

Orx had no effect on callus width and density in the analysis of microradiographes (Fig. 5E–H). Ost increased Cl.Wi.d compared to the Non-Orx group, whereas Ral decreased Cl.Wi.v compared to Orx group (Fig. 5E, F). Ost + Ral treatment decreased callus width and increased callus density at the ventral aspect (Fig. 5F, G).

After Orx, the expression of Rankl was increased, whereas other genes were not changed (Table 2). Ost treatment upregulated Opg, Alp, Er-α and Ar gene expression and Opg/Rankl ratio in comparison to the Non-Orx and Orx rats (Table 2). Under Ral treatment, the expression of most genes studied in the Orx rats were not changed, except upregulated expression of Er-α and Ar genes. Similar to the Orx-Ost and Orx-Ral groups, the expression of Er-α and Ar genes was higher in the Orx + Ost + Ral group than in the Non-Orx and Orx groups, but significantly lower than in the Orx-Ral group (Table 2).

## Discussion

In the present study, we demonstrated for the first time a beneficial effect of the combined treatment with Ost and Ral on bone healing in both sex hormone deficient female and male rats compared to monotherapies. The combined treatment improved biomechanical properties, which were supported by enhanced bone BMD, callus BMD and cortical volume, and reduced soft tissue volume, callus volume and callus area at the osteotomy site. Despite previously reported sex differences in bone healing in humans [15, 40], the combined treatment in our rat experiments resulted in a hard, dense, and more compact but better-mineralized callus in both sexes. There results indicate improved and accelerated bone healing during the repair phase, characterized by calcified callus formation, which is known to be compromised in osteoporotic bone [17, 20, 33, 38].

Similar beneficial effects of the combined Ost + Ral treatment were observed in the in vivo CT analysis of the lumbar vertebral body L4 presented in this study and were consistent with our previous findings in the same experimental animals, where non-osteotomized bone showed comparable responses [23, 25]. Other antiresorptive and osteo-anabolic agents, such as the investigational SARM S-101479 and Ral [16], as well as teriparatide and denosumab [34], have been also reported to have synergistic beneficial effects on non-fractured bone tissue. In our study, the combination of the antiresorptive Ral, which is known to reduce bone resorption and bone loss [10, 36] with the osteo-anabolic Ost, which stimulates osteoblast activity and bone formation [11] appeared to be favorable for bone healing in both experiments. Some bone parameters of both osteotomized tibia and L4 were even higher than in the healthy control rats. Thus, the combined Ost + Ral treatment effectively prevented not only bone loss due to sex hormone withdrawal in Ovx and Orx rats [5, 24], but also the age-related changes in bone [14].

The effect of Ral treatment on bone parameters was similar but less pronounced than that observed under Ost + Ral treatment. Bone biomechanical parameters, total bone and calcified callus densities, bone volume fraction were similarly increased by the Ral treatment, however soft tissue volume and callus area and width were at the level of osteoporotic rats in both experiments. The first osseous bridging in the Ral-treated groups occurred at nearly the same time as in the Ovx or Orx osteoporotic groups, indicating that Ral neither accelerated nor delayed calcified callus formation. This could be explained by the inhibitory effect of Ral on osteoclasts, resulting in reduced bone remodeling [36]. Positive effects on bone healing have been described in the literature in an Ovx rat model, similar to that used in the present study, as well as in a young male Orx model [48, 49]. In contrast, Peters-Ueno et al. [41] observed a negative effect of Ral on bone healing in an aged non-ovariectomized rat model, in which Ral was administered for 3 months prior to osteotomy but discontinued afterward. In that study, osteotomy was performed in 21-month-old rats, and healing was evaluated one and eight weeks postoperatively [41]. It should be noted that Ral is indicated for the treatment of postmenopausal osteoporosis, but its use in older osteoporotic women is uncommon due to increased thromboembolic risk and limited benefit in this age group [43]. In our study, Ral had an overall beneficial effect on bone healing, regardless of the sex of the rats. Furthermore, previously conducted ex-vivo analyses of lumbar spine and femora also demonstrated positive effects of Ral in these rats [23, 25].

Ost treatment in both experiments did not significantly alter bone parameters at the osteotomy site compared with the osteoporotic control groups (Ovx or Orx). In Ovx rats, osteotomy bridging after Ost treatment occurred later than in the Ovx-Ost + Ral group. In our previous study, a higher dose of Ost administered after osteotomy enhanced callus formation, increased callus density and accelerated osteotomy bridging in the Ovx rat model [27]. However, the uterotrophic effect of Ost was strong at that dose [27] and therefore, a lower dose was used in the present study which showed less effect on bone healing. In our previous study in Orx rats, a similar prophylactic Ost treatment for osteoporosis increased callus area and density at the osteotomy site [29]. In the present study, callus width was also higher, although this effect was observed only compared with the Non-Ovx group. Recent findings also demonstrated that another SARM, YK11, promoted the repair of cranial bone defects in 7-week-old healthy male rats [53]. In L4 of Ovx rats, Ost treatment enhanced the stress–strain index in the present study, and also improved bone parameters in ex-vivo detailed analyses of lumbar spine and femur in both Ovx and Orx rats [23, 25]. Taken together, the anabolic effect on non-osteotomized bone and the lack of negative impact on bone healing suggest that Ost may maintain bone quality under sex hormone-deficient conditions, even though its direct influence on fracture repair appears limited.

In general, Ovx in female rats was associated with more pronounced changes in callus gene expression compared to Orx in male rats. In Ovx rats, gene expression of bone markers, as well as Er-α and Ar, along with serum CTX-I levels, was higher compared to the Non-Ovx control, whereas in 8-month-old Orx rats only Rankl expression was elevated, with no consistent changes observed in other genes or serum markers. These differences may be related not only to sex hormone deficiency but also to the age of the animals. Ovx rats (6 months old) typically exhibit a high-turnover bone metabolism that may accelerate callus remodeling, whereas older Orx rats show a lower-turnover pattern [5, 24], which may contribute to slower bone healing. This is further supported by the analysis of osseous bridging at the osteotomy site, which occurred earlier in Ovx rats (around day 20) compared to Orx rats (around day 27). Bone loss had been confirmed in previous analyses of the lumbar spine and femur in both experiments [23, 25].

Within the Ost, Ral, and Ost + Ral groups, the most consistent effects on callus gene expression and serum parameters were observed under combination therapy, whereas Ost and Ral alone induced more variable and, in some parameters, sex-specific changes. In Orx male rats, Ost upregulated most genes studied, except for Oc and Rankl, although no clear improvement in bone healing was observed. In contrast, several non-fracture osteoporosis models in both sexes have demonstrated anabolic effects of Ost and other SARMs on bone structure, including increased BMD and improved trabecular architecture, although these changes were often not accompanied by biomechanical improvements [6, 19, 22]. This may indicate that SARM-induced anabolic effects are beneficial for non-fractured bone but are insufficient or differently regulated during fracture repair, where remodeling, callus formation, vascularization, and mechanobiological factors play dominant roles [4]. Ral reduced serum CTX-I levels in Orx males, confirming its bone turnover–decreasing effect [42], and additionally increased Er-α and Ar expression in the callus. Under Ost + Ral treatment, both Er-α and Ar remained elevated, although their expression levels were significantly lower than under Ral alone. Combined treatment also reduced serum OC and CTX-I levels, while increasing serum AP activity and decreasing Alp expression in the callus. These changes under combined therapy may primarily reflect the anti-resorptive effects of Ral rather than the anabolic effects of Ost [6, 42]. In Ovx female rats, treatment effects were limited to relatively few parameters. Ost increased serum AP activity but did not affect callus gene expression. Ral downregulated Oc expression, whereas Ost + Ral treatment reduced both callus Oc expression and serum OC levels while increasing Opg and Ar expression. This may reflect the substantial impact of Ovx on bone metabolism [24], which exceeded the magnitude of most treatment-induced changes. Overall, gene expression responses were more sensitive to treatment-induced changes than serum parameters, indicating stronger local than systemic effects.

In our study, Ost administrated immediately after Ovx or Orx prevented the atrophy of uterus and prostate, indicating persistent hormonal stimulation through ARs [23, 25, 46]. Ral had no effect on either organ, which can be considered as a desirable outcome. The androgenic effect of Ost on the prostate was reduced when combined with Ral [25].

Limitations of this study include the use of sex‑hormone‑deficient rat models, which do not fully replicate the complexity of human osteoporotic fracture healing, the lower Ost dose selected to avoid uterotrophic effects [27], the assessment of bone healing at a single time point, and the absence of long‑term safety evaluation.

In summary, the combined treatment with Ost and Ral enhanced bone healing in both Ovx and Orx rats, improving biomechanical properties, bone density, and accelerating callus bridging, while reducing soft tissue volume at the fracture site. These beneficial effects of osteoanabolic and antiresorptive treatments were superior to either monotherapy. Ral alone improve bone density but did not accelerate callus mineralization, and Ost maintained bone quality without markedly enhancing fracture healing at the given dose. Their complementary mechanisms, Ral providing structural stability through preservation of bone architecture, and Ost stimulating osteoblast mediated bone formation, enabled more effective repair, producing a dense, well mineralized callus with superior biomechanical strength. The observed sex-specific molecular responses highlight the influence of hormonal status and age on bone regeneration, and suggest that combination strategies targeting both bone formation and resorption may offer therapeutic potential for osteoporotic fracture healing. Overall, these findings support further investigation of selective androgen and estrogen receptor modulation as a targeted therapeutic approach, with careful evaluation of systemic safety.

## Supplementary Information

Below is the link to the electronic supplementary material.

Supplementary file1

Supplementary file2
