# Correction to: Improvement of Bone Healing by Combined Treatment with Selective Androgen and Estrogen Receptor Modulators in Female and Male Rats

**DOI:** 10.1007/s00223-026-01609-4

**Published:** 2026-10-10

**Authors:** Marina Komrakova, Fabian Nicolai Brunnert, Christian Anders, Kai Oliver Böker, Arndt Friedrich Schilling, Paul Jonathan Roch, Daniel Bernd Hoffmann, Wolfgang Lehmann, Stephan Sehmisch

**Affiliations:** 1 Department of Trauma Surgery, Orthopaedics and Plastic Surgery, University Medical Center Goettingen, Robert-Koch St. 40, 37075 Goettingen, Germany; 2 Department of Trauma Surgery, Hannover Medical School, University of Hannover, Carl-Neuberg-Str. 1, 30625 Hannover, Germany

**Correction to: Calcified Tissue International (2026) 117:132** **https://doi.org/10.1007/s00223-026-01585-9**

In the original version of this article, the given and family names of all authors were incorrectly structured. The corrected author names were provided here and the original article has been corrected.

**Incorrect Author list**: Komrakova Marina^1^ · Brunnert Fabian Nicolai^1^ · Anders Christian^1^ · Böker Kai Oliver^1^ · Schilling Arndt Friedrich^1^ · Roch Paul Jonathan^1^ · Hoffmann Daniel Bernd^1^ · Lehmann Wolfgang^1^ · Sehmisch Stephan^1,2^

**Correct Author list**: Marina Komrakova^1^, Fabian Nicolai Brunnert^1^, Christian Anders^1^, Kai Oliver Böker^1^, Arndt Friedrich Schilling^1^, Paul Jonathan Roch^1^, Daniel Bernd Hoffmann^1^, Wolfgang Lehmann^1^, Stephan Sehmisch^1,2^

